# The Profile of Immunophenotype and Genotype Aberrations in Subsets of Pediatric T-Cell Acute Lymphoblastic Leukemia

**DOI:** 10.3389/fonc.2019.00316

**Published:** 2019-04-30

**Authors:** Elda Pereira Noronha, Luísa Vieira Codeço Marques, Francianne Gomes Andrade, Luiz Claudio Santos Thuler, Eugênia Terra-Granado, Maria S. Pombo-de-Oliveira, Brazilian Collaborative Study Group of Acute Leukemia, Carolina da Paz Zampier

**Affiliations:** ^1^Pediatric Hematology-Oncology Program, Research Center, Instituto Nacional de Câncer, Rio de Janeiro, Brazil; ^2^Clinical Research Division, Research Center, Instituto Nacional de Câncer, Rio de Janeiro, Brazil

**Keywords:** T-cell acute lymphoblastic leukemia, childhood, immunophenotypic subtypes, molecular alterations, early T-cell precursor acute lymphoblastic leukemia, overall survival

## Abstract

T-cell acute lymphoblastic leukemia (T-ALL) is a biologically heterogeneous malignancy, which reflects distinctive stages of T-cell differentiation arrest. We have revisited a cohort of pediatric T-ALL, in order to test if immunophenotypes associated with molecular alterations would predict the patient's outcome. Genetic mutations, translocations and copy number alterations were identified through Sanger sequencing, RT-PCR, FISH and multiplex ligation-dependent probe amplification (MLPA). We defined 8 immunophenotypic T-ALL subtypes through multiparametric flow cytometry: early T-cell precursor (ETP, *n* = 27), immature (*n* = 38), early cortical (*n* = 15), cortical (*n* = 50), late cortical (*n* = 53), CD4/CD8 double negative mature (*n* = 31), double positive mature (*n* = 35) and simple positive mature (*n* = 31) T-ALL. Deletions (del) or amplifications (amp) in at least one gene were observed in 87% of cases. The most frequent gene alterations were *CDKN2A/B*^del^ (71.4%), *NOTCH1*^mut^ (47.6%) and *FBXW7*^mut^ (17%). ETP-ALL had frequent *FLT3*^mut^ (22.2%) and *SUZ12*^del^ (16.7%) (*p* < 0.001), while *CDKN2A/B*^del^ were rarely found in this subtype (*p* < 0.001). The early cortical T-ALL subtype had high frequencies of *NOTCH1*^mut^ and *IL7R*^mut^ (71%, 28.6%, respectively), whereas, mature T-ALL with double positive CD4/CD8 had the highest frequencies of *STIL-TAL1* (36.7%), *LEF1*^del^ (27.3%) and *CASP8AP2*^del^ (22.7%). The co-existence of two groups of T-ALL with *NOTCH1*^mut^*/IL7R*^mut^, and with *TLX3/SUZ12*^del^*/NF1*^del^/*IL7R*^mut^, were characterized with statistical significance (*p* < 0.05) but only *STIL-TAL1* (pOS 47.5%) and *NOTCH1*^WT^/*FBXW7*^WT^ (pOS 55.3%) are predictors of poor T-ALL outcomes. In conclusion, we have observed that 8 T-ALL subgroups are characterized by distinct molecular profiles. The mutations in *NOTCH1/FBXW7* and *STIL-TAL1* rearrangement had a prognostic impact, independent of immunophenotype.

## Introduction

T-cell acute lymphoblastic leukemia (T-ALL) is a biologically heterogeneous malignancy that reflects the stage of T-cell differentiation arrest. The frequency of T-ALL is about 15% of pediatric ALL cases, being characterized clinically as a predictive high-risk group for long-term outcome (1). The understanding of T-cell biology has substantially improved, and nowadays the 5-years survival rate is about 65–70% in some T-ALL settings (2). Unlike B-cell precursor ALL, in which the immunophenotype-genotype association profile is well established as outcome predictors, T-ALL still needs further investigations. The classification of T-ALL into the four immunophenotypic sub-groups, pro-T, pre-T, cortical, and mature (3), has been under scrutiny for clinical-therapeutical decisions. For instance, CD1a phenotype and CD56 were first associated with outcome (1, 4), but in further studies a null effect has been found (5, 6). The intensification of treatment through consolidation, maintenance regimen and re-intensification has led to considerable benefits to pediatric T-ALL remission rates (7). In 2009, the early T-cell precursor acute lymphoblastic leukemia subtype (ETP-ALL) was characterized by presence of lymphoblasts expressing cytoplasmic CD3 and CD7, in addition to stem cell and myeloid antigens, and the gene mutational spectrum is similar to that of poor differentiated myeloid neoplasm (8, 9). Although this entity was initially described as a predictive high-risk group, recent studies demonstrated no consensus regarding ETP-ALL diagnosis and outcome (2, 10, 11).

Recently, genomic landscape data have shown that the gene lesions frequently found in T-ALL are associated with maturational subtypes of T-ALL (12, 13). Liu et al. (12) have described 10 altered pathways according to T-ALL maturational stage. For instance, *NRAS/FLT3* mutations were associated with immature T-ALL (12). It is expected that the genomic landscape provides a logical framework for new therapeutic approaches, therefore, it is very important to establish an algorithm of tests that could quickly predict genetic lesions at diagnosis. Even though T-ALL outcomes have significantly improved in patients that received intensive treatment protocol, unveiling the associations between immunophenotypic profile and genetic abnormalities in T-ALL can be of great importance to establish subsets with distinct prognostic relevance, providing means to avoid late toxicity through risk adaptation of treatment with target therapy. Here, we have revisited a series of pediatric T-ALL and tested if immunophenotypic profiles are associated with distinct molecular alterations and if these associations would predict patient's outcome.

## Materials and Methods

### Patient Samples

Three hundred forty-one T-ALL cases (age <19 years), sent to the Pediatric Hematology-Oncology Program, Research Center, Instituto Nacional de Câncer, Rio de Janeiro, Brazil, for diagnostic tests (2005–2017), were reviewed in this study. The study design with selection criteria is shown in Supplementary Figure 1. Viable frozen cells were available to complete the immunophenotyping panel according to ETP-ALL criteria from Inukai et al. (11) and to perform additional molecular tests. Cases that had the diagnosis of lymphoblastic lymphoma with <20% of bone marrow infiltration (*n* = 12) were excluded from this study. Myeloid/T-cell mixed phenotype acute leukemia (*n* = 10), samples with low cell viability (*n* = 11) and samples not tested for cytoplasmic CD3, membrane CD3 and/or CD1a (*n* = 26) were also excluded.

The referring physicians provided information about the demographic and clinical follow up data for the patients. Patients were not formally enrolled in treatment protocols, but were treated according to either the Brazilian Group for Treatment of Childhood Leukemia (GBTLI-ALL99) or the Berlin-Frankfurt-Munster ALL (BFM–ALL) backbone protocols (14, 15).

The treatment outlines were similar, as in the induction phase, all patients received a pre-phase of prednisone (7 days) and intrathecal dose of Methotrexate (MTX). The induction phase lasted 4 weeks, and included prednisone, Vincristine, doxorubicin, L-asparaginase, and intrathecal MTX, Cytarabine and dexamethasone treatment. All patients received adequate treatment for prevention of CNS relapse with chemotherapy. Children with CNS infiltration at the diagnosis received cranial radiotherapy.

### Ethics

This study was carried out in accordance with the recommendations of Instituto Nacional de Câncer Research and Ethics Committee. Written informed consent from the parents or legal guardians was obtained from children and adolescents. All young subjects gave written informed consent in accordance with the Declaration of Helsinki. The protocol was approved by the Instituto Nacional de Câncer Research and Ethics Committee under the registry number CEP/INCA#117/12; CONEP: PB #888.2772.3.

### T-ALL Immunophenotypic Characterization

In all cases, the immunophenotyping by multiparametric flow cytometry was performed utilizing the panel of monoclonal antibodies in Supplementary Table 1. FACS Calibur and FACS Canto II flow cytometers (Becton, Dickinson, and Company, CA, USA) were used for the sample acquisition and all the immunophenotypic analyses were performed in the Infinicyt™ program version 1.8 (Cytognos—Salamanca—Spain), according to previously published procedures (16, 17). A sample was considered positive for a marker when at least 20% of lymphoblasts in a CD45^low^/^intermediate^ gate had its expression.

The immunophenotypic classification of T-ALL subtypes was applied using previously published criteria (3, 8, 11). Immunophenotyping was performed by 6 color combination of monoclonal antibodies, applying the Coustan-Smith et al. (8) criteria with the addition of the score system from Inukai et al. (11) to identify ETP-ALL cases.

### T-ALL Molecular Characterization

Diagnostic samples from T-ALL were first subjected to total RNA extraction using TRIzol (Life Technologies, Grand Island, NY, USA) according to the manufacturer's instructions. Complementary DNA (cDNA) was synthesized using 2 μg of total RNA with the First-Strand cDNA Synthesis Kit™ (Amersham Pharmacia Biotech, Amersham Biosciences UK Limited, Little Chalfont/UK); cDNA integrity was examined by amplifying a fragment of the glyceraldehyde-3-phosphate dehydrogenase (*GAPDH*) constitutive gene, accordingly. The *STIL-TAL1* and *TX3* rearrangement were investigated by Reverse Transcriptase-PCR, as previously published (18). Partial clinical, molecular and prognostic data on Brazilian samples diagnosed from 2005-2012 have been previously published (19, 20).

Genomic DNA from the same leukemic cell samples was also obtained using a QIAamp® DNA Blood Mini Kit (Qiagen GmbH, Hilden, Germany), as recommended by the manufacturer. Mutations were investigated in the hotspot regions of the following genes: *NOTCH1, FBXW7, IL7R, RAS* (*NRAS* and *KRAS*), and *FLT3. NOTCH1* mutations were analyzed by screening of the heterodimerization (HD) (exon 26 and 27) and polypeptide enriched in proline, glutamate, serine and threonine (PEST) (exon 34) domains (19, 21). To evaluate the mutational status of *FBXW7*, we screened exons 9 and 10 (22); mutations in *IL7R* were investigated in exon 6 (23); mutations in *FLT3* were investigated at the tyrosine kinase domain (TKD) in codon 835 and the juxtamembrane domain in exons 11/12 as internal tandem duplications (ITD) and *N/KRAS* status was determined by searching mutations in exon 1 (codons 12/13) (24). All PCR products were purified, and Sanger sequencing was performed using a BigDye Terminator v3.1 Cycle Sequencing Kit (Applied Biosystems, Carlsbad, CA) in a 3,500 Genetic Analyzer (Applied Biosystems). The analyses were performed with BioEdit 7.0.9 software, comparing electropherograms with the reference sequences accessed from the National Center for Biotechnology Information (NCBI): *NOTCH1* (NG_007458.1; NM_017617.3), *FBXW7* (NM_1013415.1; NG_029466.1), *IL7R* (NG_95671.1; NM_2184.3), *KRAS* (NG_7524.1; NM_004985.4) and *NRAS* (NM_002524.4; NG_007572.1).

The multiplex ligation-dependent probe amplification (MLPA) was performed using the SALSA MLPA probe mix P383-A1 TALL (MRC Holland, Amsterdam, The Netherlands) to identify copy number alterations (CNA). This kit is able to assess alterations (deletions or amplifications) in transcription factors (*LEF1* and *MYB*)*;* in signal transduction (*PTEN, NF1* and *PTPN2*); in cell cycling (*CDKN2A, CDKN2B* and *CASP8AP2)* and in epigenetic regulator genes (*EZH2, SUZ12* and *PHF6*) and identify the *STIL-TAL1* and *NUP214-ABL1* fusion genes. The procedure and analyses of data were done according to the manufacturer's instructions. Analyzes was done in Coffalyser software v.140721.1958 (MRC-Holland, Amsterdam, The Netherlands).

### Statistical Analyses

To compare the distribution of demography, clinical variables, cellular and molecular alterations between T-ALL subtypes, we have used the χ2 test. Univariate *p* values were calculated using Pearson's chi-square test or Fisher exact test. Two-sided *p*-values with a significance limit of <0.05 were considered throughout the study. The frequency of genetic aberration and analysis of concomitances were illustrated by Circos plots (25).

The probability of overall survival (pOS) in 60 months was determined using the Kaplan–Meier method in months from the diagnosis to the outcome (death, survival or last follow-up). Patients who lost to follow-up were censored at their date of last known information. The differences between T-ALL survival distributions were compared by the log-rank test. The multivariate Cox proportional hazard regression method was used to determine the independent prognostic factors influencing pOS. Multivariate Cox analysis was performed with variables associated with a *p* < 0.2 in univariate analysis. To compare the immunophenotypic profiles regarding demographic, clinical and molecular characteristics, and OS each subtype vs. the remaining subtypes together were considered.

SPSS (Statistical Product and Services Solutions, version 18.0, SPSS Inc, Chicago, IL, USA) software was used for data analyses.

## Results

The T-ALL cases previously characterized according to EGIL classification [T-I (*n* = 7), T-II (*n* = 73), T-III (*n* = 103), and T-IV (*n* = 99)] were revisited, and with the addition of CD117, CD11b, and CD15 analysis, they were re-classified according to Inukai et al. (11) score system (Supplementary Figure 2). Twenty-seven cases previously classified as T-I (*n* = 7) and T-II (*n* = 20) were re-classified as ETP-ALL, representing 9.7% of the total T-ALL cohort. The remaining cases were subdivided as follows: (1) immature T-ALL (CD2^pos^ and/or CD5^pos^ and/or CD8^pos^ or CD4^pos^; *n* = 38); (2) early cortical (CD4^pos^ and CD8^pos^, CD1a^neg^, mCD3^neg^; *n* = 15); cortical (CD1a^pos^ and mCD3^neg^; *n* = 50); late cortical (CD1a^pos^ and mCD3^pos^; *n* = 53); and (3) mature T-ALL, (CD1a^neg^ and mCD3^pos^; *n* = 99). The latter was subdivided in CD4/CD8 double positive (DP) (*n* = 35), single positive (SP) either CD4^neg^/CD8^pos^ or CD4^pos^/CD8^neg^ (*n* = 31) and CD4/CD8 double negative (DN) (*n* = 31). All T-ALL cases were cCD3 and CD7 positive. The demographic and clinical features were analyzed according to reviewed T-ALL subtypes and shown in Table 1. ETP-ALL subtype was associated with patients older than 10 years at the diagnosis and circulating white blood cell count (WBC) of <50 × 10^9^/L (*p* = 0.02 and 0.042, respectively). No significant differences were found in demographic and/or clinical features according to immature, early and late cortical and/or mature T-ALL subtypes (Supplementary Table 2).

**Table 1:** Demographic, clinical-laboratorial and molecular features of pediatric T-ALL in Brazil, 2005–2017.

**Variables**	**Total *n* (%)**	**ETP-ALL *n* (%)**	**T-ALL *n* (%)**	***p*-value**
**AGE (YEARS)**				
<1	7 (2.5)	2 (7.4)	5 (20)	0.029
1–9	153 (54.3)	9 (33.3)	144 (56.5)	
≥10	122 (43.3)	16 (59.3)	106 (41.6)	
**SEX**				
Female	72 (25.5)	10 (37)	62 (24.3)	0.166
Male	210 (74.5)	17 (63)	193 (75.7)	
**WBC (×10**^**9**^**/L)**				
<50	81 (28.7)	13 (48.1)	68 (26.7)	0.042
50–100	53 (18.8)	2 (7.4)	51 (20.0)	
≥100	148 (52.5)	12 (44.4)	136 (53.3)	
**MEDIASTINAL MASS**				
Yes	114 (40.7)	7 (25.9)	107 (42.0)	0.230
No	166 (58.9)	20 (74.1)	146 (57.3)	
Missing	2 (0.7)	–	2 (0.8)	
**CNS INFILTRATION**				
Yes	19 (6.7)	2 (7.4)	17 (6.7)	0.891
No	261 (92.6)	25 (92.6)	236 (92.5)	
Missing	2 (0.7)	–	2 (0.8)	
**LYMPH NODE INFILTRATION**				
Yes	195 (69.1)	20 (74.1)	175 (68.6)	0.942
No	85 (30.1)	7 (25.9)	78 (30.6)	
Missing	2 (0.7)	–	2 (0.8)	
**FOLLOW UP STATUS**				
Alive	213 (75.5)	18 (66.7)	195 (76.5)	0.249
Dead	69 (24.5)	9 (33.3)	60 (23.5)	
Total	282 (100)	27(9.5)	255 (90.4)	

*n, number of cases; WBC, White blood cell count; CNS, central nervous system*.

The frequencies of molecular alterations in overall cases and according to revisited T-cell subtypes are shown in Table 2. *NOTCH1* mutations were found in 110 out of 231 (47.6%) cases; among these *NOTCH1*^mut^ cases, 62 (56.4%) had solely HD domain mutations, 27 (24.5%) in the PEST/TAD domain, and 21 (19.1%) had mutations in both HD and PEST domains (data not shown). Thirty-eight cases had *FBXW7*^mut^ (17%), 19 *N/KRAS*^mut^ (8.2%), 16 *IL7R*^mut^ (7.1%), and 9 cases *FLT3*^mut^ (4.1%). The *STIL-TAL1* fusion gene was found in 49 cases (21.2%) and 25 cases were positive for *TLX3* (10.2%), which were mutually exclusive. The co-occurrence of both *NOTCH1* and *FBXW7* mutations were found in 129 cases (57.6%). A heterogeneous distribution of frequencies of these molecular alterations was observed among the T-ALL subtypes, although few specific molecular alterations were associated with the immunophenotyping. The frequencies of *NOTCH1*^mut^ varied from 37% to 71.4% across the T-ALL subtypes. The early cortical T-ALL presented the highest frequencies of *NOTCH1*^mut^ and *IL7R*^mut^ (71.4% and 28.6%, respectively; *p* = 0.01). *FLT3*^mut^ was most frequent in the ETP-ALL subgroup (*p* < 0.001), whereas *IL7R*^mut^ in early cortical T-ALL and *N/KRAS*^mut^ in mature DN T-ALL (*p* < 0.05). *IL7R*^mut^ was absent in ETP-ALL, and *N/KRAS*^mut^ in late cortical and DP mature T-ALL (Table 2). Some molecular alterations were age-associated: 16 out 19 (84.2%) of T-ALL with *N*/*KRAS*^mut^ were found in children <10 years old (*p* = 0.037).

**Table 2:** Molecular alterations according to pediatric T-cell Acute Lymphoblastic Leukemia subtypes, Brazil 2015–2017.

							**Mature**		
**Molecular alterations**	**n/nt (%)**	**ETP n/nt (%)**	**Immature n/nt (%)**	**Early cortical n/nt (%)**	**Cortical n/nt (%)**	**Late cortical n/nt (%)**	**DP n/nt (%)**	**SP n/nt (%)**	**DN n/nt (%)**
*STIL-TAL1*^pos^	49/231 (21.2)	3/24 (12.5)	5/33 (15.2)	2/10(20)	6/37 (16.2)	10/44 (22.7)	11/30 (36.7)	8/25 (32)	4/26 (15.4)
*TLX3*^pos^	25/245 (10.2)	2/23 (8.7)	1/32 (3.1)	2/11 (18.2)	4/42 (9.5)	6/46 (13)	2/31 (6.5)	3/29 (10.3)	5/29 (17.2)
*NOTCH1*^mut^	110/231 (47.6)	10/27 (37)	13/29 (44.8)	10/14 (71.4)	23/39 (59)	22/43 (51.2)	12/30 (40)	8/22 (36.4)	11/24 (45.8)
*FBXW7*^mut^	38/224 (17)	3/27 (11.1)	5/30 (16.7)	3/14 (21.4)	9/38 (23.7)	8/43 (18.6)	4/27 (14.8)	4/20 (20)	2/23 (8.7)
*N/KRAS*^mut^	19/231 (8.2)	3/27 (11.1)	3/31 (9.7)	3/13 (23.1)	3/38 (7.0)	0/45 (0)^*^	0/28 (0)	1/24 (4.2)	6/23 (26.1)^**^
*IL7R*^mut^	16/225 (7.1)	0/26 (0)	1/31 (3.2)	4/14 (28.6)^*^	2/38 (5.3)	6/42 (14.3)	2/28 (6.7)	0/20 (0)	1/22 (4.5)
*FLT3*^mut^	9/222 (4.1)	6/27 (22.2)^***^	0/28 (0)	0/14 (0)	1/38 (2.6)	2/43 (4.7)	0/30 (0)	0/18 (0)	0/22 (0)

n. number of cases with alterations; nt, total of cases tested; pos, positive; mut, mutated;

* p < 0.05;

** p < 0.01;

*** *p < 0.001*.

One hundred and sixty-eight T-ALL cases had a good quality of available DNA to perform MLPA tests. To test the possible selection bias, we have compared the demographic and clinical characteristics, T-ALL subsets and molecular aberrations of these 168 cases tested (Supplementary Table 3). There were equal frequency distributions of all variables among the selected cases tested, but there was a decreased number of cases with WBC lower than 100 × 10^9^/L among non MLPA tested T-ALLs (*p* < 0.01).

**Figure 1:**
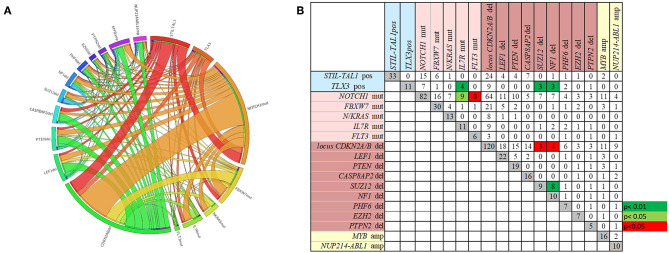
The frequencies and the concomitance of molecular alterations in 168 pediatric T-ALL cases. **(A)** Circos plot showing the overall co-occurrence of mutations, rearrangements, and CNA assessed through MLPA. Outer segments proportionally represent the alterations found in T-ALL. Interior lines connecting the outer segments proportionally demonstrate the concomitances among genetic alterations. **(B)** Number of cases with each molecular alteration in gray squares, and number of cases with concomitances between two alterations in white, green and red squares. Green squares represent positive while the red squares negative associations. Alterations ordered by type of abnormality. Rearrangements in blue, mutations in pink, CNA deletions in brown and amplifications in yellow.

The overall frequencies and the concomitance of gene alterations in 168 T-ALL cases are shown in Figures 1A,B. Deletions (del)/amplifications (amp) in at least one gene was identified in 87% of cases. The most frequent genomic aberration was *CDKN2A/B*^del^ found in 71.4% of the cases, with a predominant biallelic status (78.3%). *LEF1*^del^ (13%), *PTEN*^del^ (11.3%) and *CASP8AP2*^del^ (9.5%) were also recurrent. The most frequent gene amplifications were found in *MYB* (9.5%), and *NUP214-ABL1* fusion gene was identified in 6% of cases. The *IL7R*^mut^ (*n* = 16) was found associated with *NOTCH1*^mut^ (*n* = 13) and *TLX3*^*pos*^ (*n* = 5) (*p* < 0.01). Likewise, *NF1*^*del*^ and *SUZ12*^*del*^ were found concomitantly in three out of eleven *TLX3*^pos^ cases (*p* < 0.01). On the other hand, co-occurrence of *NF1*^*del*^ and *SUZ12*^*del*^ with *CDKN2A/B*^del^ was rarely observed (*p* < 0.05). In summary, the molecular concomitance reflects the T-ALL heterogeneity, and two groups were well defined: one with *NOTCH1*^mut^*/IL7R*^mut^, and another with *TLX3*^*pos*^*/SUZ12*^del^*/NF1*^del^*/IL7R*^mut^ (Figure 1; *p* < 0.05).

**Figure 2:**
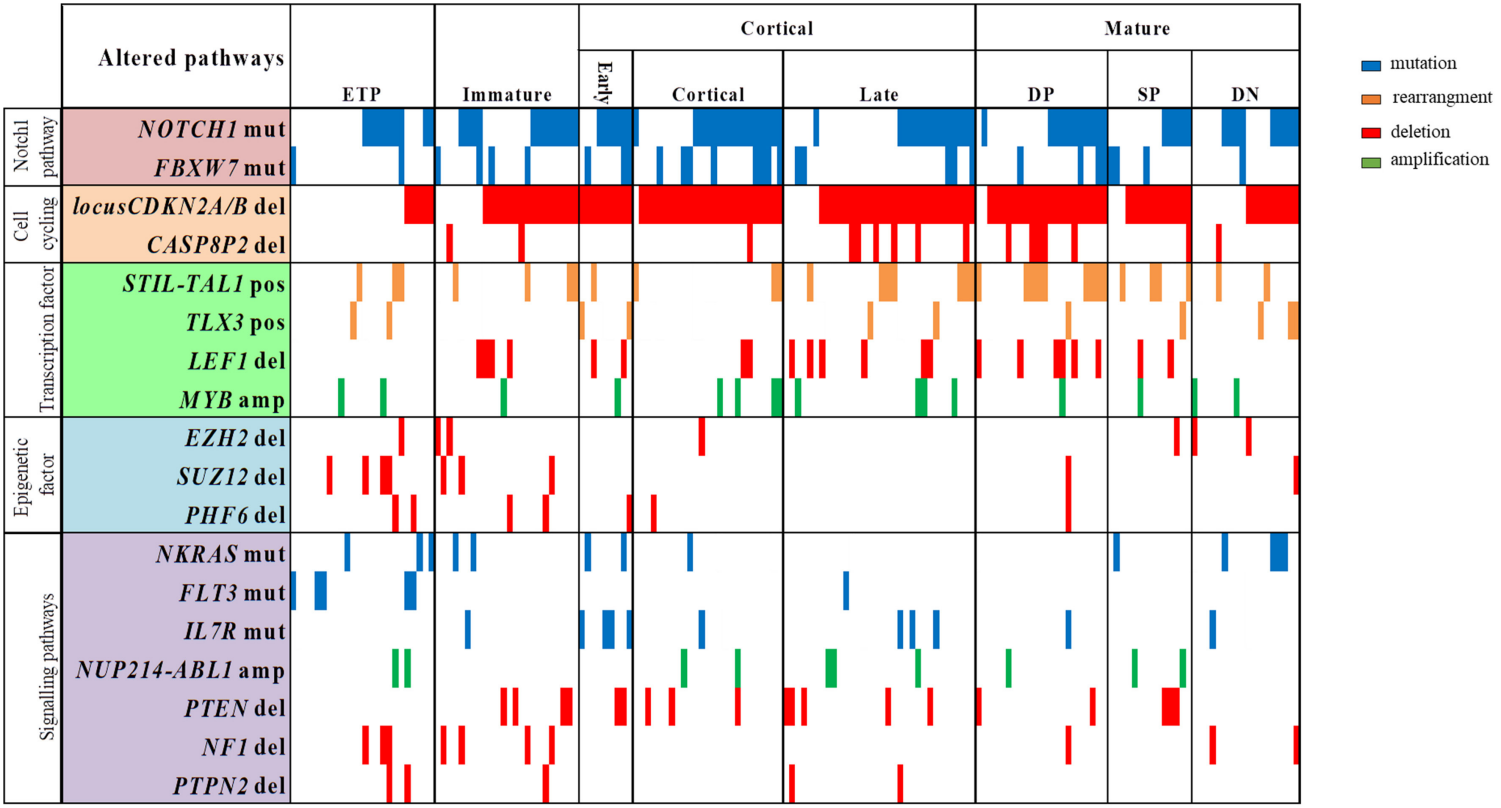
Overview of molecular alterations identified in 168 samples according to T-ALL subtypes and grouped into recurrently altered pathways. ETP, early T cell precursror; DP, douple positive for CD4/CD8; SP, single positive for CD4/CD8; DN, double negative for CD4/CD8; mut, mutation; del, deletion; pos, positive; amp, amplification. Vertical lines represent each patient.

The profile of T-ALL subtypes and the coexistence of molecular alterations, according to genes with functions as transcription factors, cell cycling regulator, cell signaling pathways and epigenetic regulators, are better visualized in the Figure 2. We have observed that ETP and immature T-ALL subtypes were enriched with alterations in epigenetic regulators *EZH2, SUZ12*, and *PHF6* (*p* < 0.001 and *p* = 0.02, respectively), whereas these alterations were less frequently found in the late cortical and mature T-ALL subtypes (*p* = 0.013). ETP-ALL subtype has the highest frequencies of *SUZ12*^del^ (16.7%; *p* = 0.025), while *CDKN2A/B*^del^ (*p* < 0.001), *CASP8AP2*^del^, *LEF1*^del^, *PTEN*^del^, and *IL7R*^mut^, were not found in this subtype. *CDKN2A/B*^del^ was present in all early-cortical T-ALL subtype, cortical (96%) and mature DP subtype (91%), (*p* < 0.01; *p* = 0.040, respectively). Mature DP T-ALL subtypes have the highest frequencies of *LEF1*^del^ and *CASP8AP2*^del^ (27.3 and 22.7%, respectively, *p* = 0.04; *p* = 0.046).

The results of the univariate analysis for pOS of T-ALL cases, regarding clinical, immunophenotypic subtypes and molecular alterations are shown in Supplementary Table 4. The median time of OS of the whole cohort was 42.7 months (95%CI: 39.5-46.2; 5-years pOS 62% ± 0.04). No significant difference in pOS was found for the ETP-ALL when compared to other T-ALL subtypes, WBC level, sex and central nervous system (CNS) infiltration. However, patients with an age <12 months-old had a lower pOS (22.2%; *p* = 0.036) and the presence of lymph node infiltration and mediastinal mass had higher pOS (68.8%,) compared to those without (49.2%; *p* = 0.008 and 0.04, respectively), and infiltration in CNS was 57.9%. Regarding molecular alterations, the pOS rates were not significant for *TLX3*^pos^, *IL7R*^mut^, *RA*S^mut^ and CNAs. The variables with a *p* < 0.2 according to univariate analysis are demonstrated in Table 3.

**Table 3:** Pediatric T-cell acute lymphoblastic leukemia and overall survival in univariate analysis, 2005-2017, Brazil.

**Variables**	**pOS % (SE)**	Mean^*^ (SE)	**95%CI**	***p*-value**
**AGE (YEARS)**				
<1	22.2 (0.19)	22.0 (9.58)	(3.2–40.8)	0.036
1–10	66.7 (0.52)	45.3 (2.23)	(40.9–49.7)	
≥ 10	58.3 (0.57)	40.9 (2.62)	(35.9–46.1)	
**LYMPH NODE INFILTRATION**				
Yes	68.8 (0.44)	45.9 (1.95)	(42.1–49.8)	0.008
No	49.2 (0.69)	36.8 (3.20)	(30.6–43.1)	
**MEDIASTINAL MASS**				
Yes	70.8 (0.56)	47.5 (2.37)	(42.8–52.1)	0.044
No	56.7 (0.50)	40.1 (2.33)	(35.5–44.7)	
**T-ALL SUBTYPES**				
Immature vs	78.5 (0.09)	49.8 (4.10)	(41.8–57.8)	0.109
Others	59.5 (0.04)	41.9 (1.84)	(38.3–45.5)	
Mature SP vs	49.4 (0.13)	36.8 (5.73)	(25.6–48.1)	0.194
Others	63.5 (0.04)	43.6 (1.77)	(40.2–47.1)	
**MOLECULAR ALTERATIONS**				
*NOTCH1*^mut^*/FBXW7*^mut^	67.6 (0.55)	46.3 (2.33)	(41.8–50.9)	0.027
*NOTCH1^*WT*^/FBXW7*^WT^	55.3 (0.62)	37.8 (3.08)	(31.8–43.8)	
*STIL/TAL1*^pos^	47.6 (0.90)	33.6 (4.51)	(24.8–42.5)	0.006
*STIL/TAL1*^neg^	66.0 (0.47)	45.4 (2.00)	(41.5–49.3)	
*MYB* ^amp^	46.7 (0.17)	31.5 (8.84)	(14.2–48.8)	0.113
*MYB*^WT^	64.4 (0.51)	43.7 (2.30)	(39.2–48.2)	
Total	62.0 (0.04)	42.8 (1.70)	(39.5–46.1)	

*pOS, probability of overall survival; SE, standard error, CI, confidence interval; SP, CD4 and/or CD8 simple positive; mut, mutated; WT, wild type; pos, positive; neg, negative; amp, amplified*.

* *mean survival in months*.

**Figure 3:**
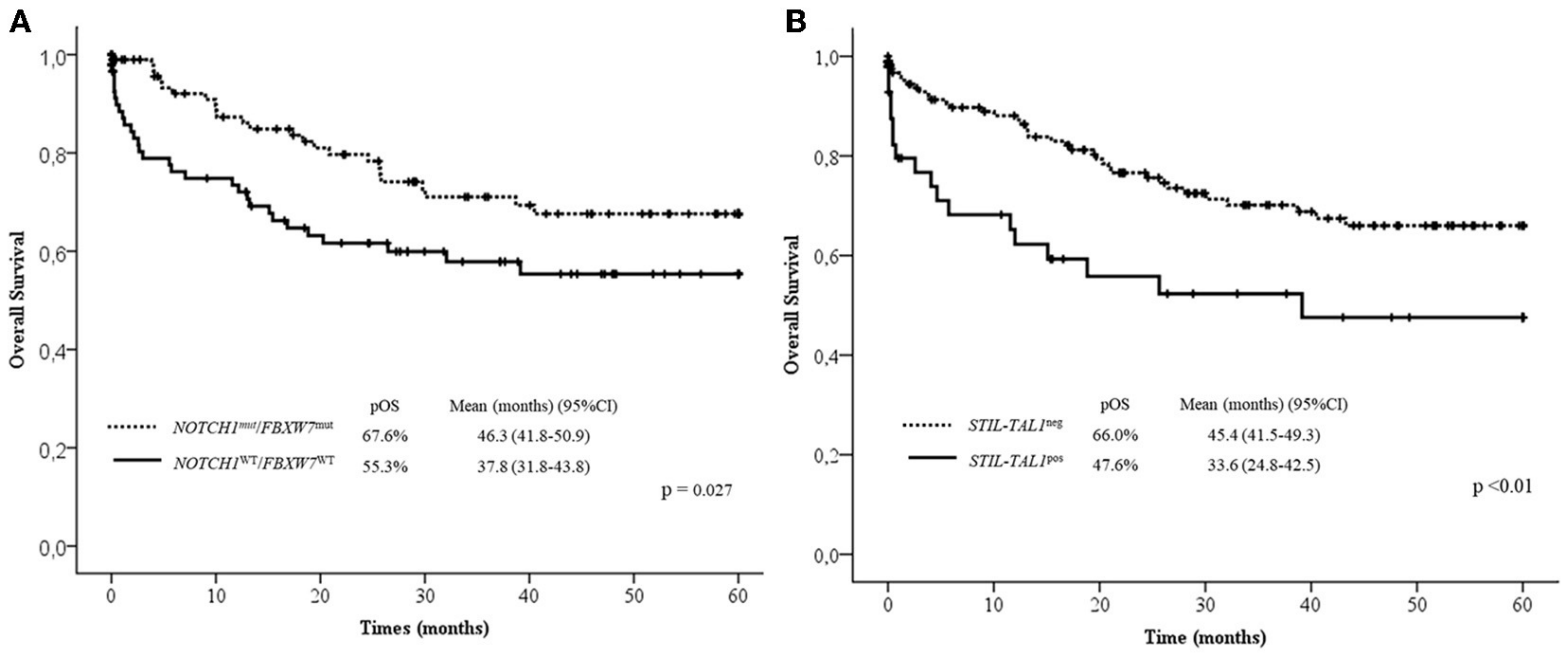
Overall survival curves in 60 months according to *NOTCH1/FBXW7* **(A)** and *STIL-TAL1* **(B)** status. Mut, Mutated; WT, Wild Type; pOS, probability of overall survival; CI, confidential interval; pos, positive.

In the multivariate analysis model shown in Table 4, the absence of lymph node infiltrations, *NOTCH1*/*FBXW7*^*WT*^ and *STIL-TAL1*^pos^ were independent risk factors to predict low pOS. Cases with *NOTCH1* and/or *FBXW7*^mut^ were associated with better pOS than *NOTCH1*^WT^ and *FBXW7*^WT^ cases. The presence of *STIL-TAL1* fusion genes was predictive of worse outcome as shown in Figure 3.

**Table 4:** Cox regression model of the overall survival variables of pediatric T-cell acute lymphoblastic leukemia, Brazil, 2005–2017.

**Variables**	**HR**	**CI 95%**	***p*-value**
Age (<1 years)	2.51	0.89–7.10	0.083
^*^Lymph node (Absence)	2.04	1.14–3.65	0.017
Mediastinum mass (Absence)	1.69	1.01–2.83	0.047
Non–immature T–ALL	2.07	0.83–5.15	0.117
Mature SP T–ALL	1.59	0.79–3.20	0.198
^*^NOTCH1 and/or *FBXW7* (WT)	2.20	1.21–3.65	0.010
^*^STIL–*TAL1*^pos^	2.34	1.25–4.38	0.008
*MYB*^amp^	2.11	0.82–5.41	0.122

*HR, hazard ratio; CI, confidence interval; SP, CD4 and/or CD8 single positive; WT, wild type; pos, positive; amp, amplified*.

* *Independent prognostic variables*.

## Discussion

Since T-ALL can be arrested at a variety of T-cell development stages, with common phenotype aberrations, we can conclude that, although important, the current EGIL definition of T-ALL is no longer able to predict genotype abnormalities or patient outcome (5, 12, 13). We have revisited the immunophenotyping of a large cohort of pediatric T-ALL to compare the subtypes with distinctive genomic alterations, implementing a new subdivision based on key differentiation markers, to validate the Inukai et al. (11) score system and to distinguish ETP-ALL among immature T-ALL (8, 11, 12, 26). ETP-ALL has emerged from genomic approaches and was correlated with immunophenotypic profile that has myeloid markers as a differentiating category, in contrast to other T-ALL subtypes. The revisited immunophenotyping demonstrated the value of CD1a, cytoplasmatic CD3, CD5 and the inclusion of myeloid markers (CD117, CD11b, CD15), not previously included in other studies, in the identification of ETP-ALL cases. All cases previously classified as pro-T (T-I) in the EGIL criteria and some of pre-T (T-II) cases were now categorized as ETP-ALL.

In the normal T-cell development process, the commitment to T-cell lineage is characterized by a gradual decrease of CD34, CD44, acquisition of CD1a and, as a later T-cell developmental event, the loss of CD1a (27). Recently, we have demonstrated the aberrant antigenic pattern of CD44 in T-ALL according to T-ALL cell subtypes. CD44 expression in mature subtypes seems to be influenced by genomic alterations in NOTCH1 signaling pathway validating the studies performed in an animal model and/or in co-cultures of human cell lines (16, 27). In the cortical stage, double positivity for CD4/CD8 was found in the majority of T-ALL cases, while the mature T-ALL subtypes were carefully subdivided according to CD4 and CD8 expression (DP, SP and DN) (28, 29). This approach allowed us to identify, by flow cytometry, the immature and mature subtypes of T-ALL with significant association with genomic abnormalities. Overall, 87% of the patients tested by MLPA for CNA have harbored genetic aberrations (either deletions or amplifications) in different frequency rates, according to the level of maturity of the T-cell leukemia. The frequencies of recurrent genetic mutations and rearrangements in our whole T-ALL cohort were in agreement with other clinical data described in the literature, with *CDKN2A/B* deletions, *NOTCH1* mutations and *STIL-TAL1* gene fusion being the more prevalent alterations (12, 13, 30). The frequencies of *NOTCH1* mutations in our series (47.6%) were similar to frequencies described in Asian patients (31, 32) and slightly lower than other studies in European and North American cohorts (52.2 to 56%), (21, 33, 34). It is important to highlight that these studies, such as ours, screened *NOTCH1* mutations in hot spot exons (26, 27 and 34). High frequencies, such as 79% described by Liu et al. (12), can be found when whole-exome sequencing is used. Additionally, the frequencies of *MYB* and *NUP214-ABL1* amplifications were also in accordance with previous studies (35, 36).

The molecular profile of ETP-ALL cases was associated with *FLT3* mutations, and enriched with alterations in epigenetic regulators (*EZH2, SUZ12*, and *PHF6*). Inactivation-associated epigenetic alterations in hematopoietic progenitors were established as arresting T-cell development and directly leading to aberrant upregulation of early hematopoietic programs transcription in ETP-ALL mouse model (37, 38). For instance, *EZH2* and *RUNX1* deletions were shown to increase RAS pathway-associated transcription (38), possibly creating a transcriptional environment susceptible to additional hits, such as *FLT3*-ITD and RAS pathway mutations, which further increases proliferation.

Recently, the role for *PHF6* was described in hematopoietic stem cell homeostasis and T-ALL leukemia initiating cell self-renewal, characterizing *PHF6* mutations as early events and drivers of leukemia stem cell activity in the pathogenesis of T-ALL (39). Although alterations in *PHF6* are often seen in ETP-ALL (9), they can also occur in more mature T-ALL subtypes (40), as observed in our cohort. In addition, the presence of deletions in *CDKN2A/B, PTEN* and *LEF1* were rarely observed in the ETP-ALL cases. On the other hand, mature T-ALL with CD4/CD8 DP has presented a high frequency of deletions in *LEF1, CASP8AP2, CDKN2A/B* genes and the presence of *STIL-TAL1* gene fusion. The mature T-ALL SP to CD4 or CD8 had low *NOTCH1* mutations and DN mature T-ALL was associated with an increased frequency of *N/KRAS* mutations. These differences highlight that these subtypes can represent distinct biological subsets and should not be classified as a single subtype (as only mature), without considering the expression level of CD4 and CD8 antigens.

The molecular aberration profile of ETP-ALL in our cases is partially in accordance with previous publications of T-ALL genomic landscapes (9, 12). However, in this subtype, *IL7R*^mut^ was not observed and the frequency of *NOTCH1*^mut^ did not differ significantly from other subtypes. The great majority of T-cell development research has been performed in animal model or in co-cultures of human cell lines. For instance, in murine thymocyte progenitors, the altered expression of *LMO2* and/or *BCL11B* were shown to cooperate with *IL7R* mutations before the CD4/CD8 double-negative stage (DN2) and led to the development of ETP-ALL (41). In another murine model, the presence of *IL7R* mutation, together with an intracellular active form of NOTCH1 (ICN1), led to the development of an aggressive T-ALL with CD4/CD8 DP profile (42, 43). The progression from DN to DP CD4/CD8 stage is characterized by thymocyte expansion, regulated by IL-7R and NOTCH1 signaling as well as *LEF1*, the transcription factor that is indispensable for thymocyte maturation (44). These *in vivo* studies might reflect our findings, since most T-ALL cases characterized as CD4/CD8 DP had *IL7R*^*mut*^ and *NOTCH1*^mut^ (81.2%), while *CDKN2A/B* and *LEF1* deletions by contrast, were rarely found in our ETP-ALL cohort.

One of our aims was to test if any molecular alterations were associated with T-ALL maturation arrest and could predict the outcome. We have first analyzed the impact of the classic prognostic factors such as age strata, sex, increased WBC and CNS infiltration. Two clinical factors presented significantly poor outcome: children with an age <1 year at the diagnosis of T-ALL and the absence of adenomegaly. We have previously tested the association of molecular alterations with both maturational subtype (ETP-ALL vs. more mature T-ALL) and the outcome. Among the subtypes identified in our cohort, the immature subtype had a reasonably good 5-years pOS (75.4%), although not statistically significant. In contrast, the ETP-ALL had a 5-year pOS of 56.7%. Recently we demonstrated that ETP-ALL with *NOTCH1*^*mut*^ was associated with significantly better pOS (90%) than *NOTCH1*^wt^ (pOS 37%; *p* = 0.017) (45). Interestingly, although the early cortical subtype had higher frequencies of *NOTCH1*^mut^, it presented a relatively low pOS (48.6%). In addition, this subtype was associated with high frequencies of *IL7R*^mut^ and *CDKN2A/B*^del^. In a large cohort of children with T-ALL, Schrappe et al. (46) reported that MRD status classified as standard, intermediate or high, allowed for discrimination of prognostic subgroups associated with T-ALL maturational stage. The cortical T-ALL corresponded to the MRD standard patients, having a better event free-survival, which led to the affirmation that the outcome differed by maturational stage (46). The genetic basis of these results could be a specific pathways genetic mutation underlying the T-ALL MRD status and outcome, for instance, *CDKN2A/B*^del^ and *NOTCH1*^*mut*^.

We found a high frequency of *CDKN2A/B* deletions in all T-ALL subtypes, except ETP-ALL, although this genetic lesion had a null effect in the pOS. Two groups of co-occurring aberrations, such as *NOTCH1*^*mut*^*/IL7R*^*mut*^ and *TLX3*^*pos*^*/SUZ12*^*del*^*/NF1*^*del*^*/IL7R*^*mut*^ were characterized with statistical significance. These genes are implicated in the pathogenesis of T-ALL in cortical and in immature subtypes. The screening of these alterations would be important to drive the selection of target therapy.

The multivariate analysis shows that *NOTCH1*^WT^ and *FBXW7*^WT^, *STIL-TAL1*^pos^ and the absence of lymph node enlargement are the most relevant indicators of inferior pOS, despite age, T-ALL subtypes and WBC.

Despite the lack of unique protocol applied, all patients have received adequate treatment for prevention of CNS relapse with poly-chemotherapy, and children with high WBC and CNS infiltration at the initial diagnosis received cranial radiotherapy (15). Therefore, our cohort study has confirmed, that the presence of *STIL-TAL1* is predictive of poor outcome, while *NOTCH1/FBXW7* mutations have the opposite effect, reinforcing the idea that the prognostic impact of *NOTCH1* and *FBXW7* seems not to be dependent on the treatment protocol applied, despite contrary studies.

Our data support the premises that genetic lesions are associated with T-ALL immunophenotypic profiles and that identifying molecular aberrations is relevant as it would allow patients to receive novel treatment agents, such as target therapies, as the front line treatment.

## Conclusion

In conclusion, our results show 8 T-ALL subgroups identified by flow cytometry. These subsets are characterized by distinct molecular profiles, as ETP-ALL and mature T-ALL subtypes, classified according to CD4 and CD8 expression. Nevertheless, immunophenotypic subtypes, classified based on T-cell differentiation, was not predictive for outcome. Of the molecular alterations, only mutations in *NOTCH1/FBXW7* and *STIL-TAL1* rearrangement had a prognostic impact independent of immunophenotype.

## Ethics Statement

This study was carried out in accordance with the recommendations of Instituto Nacional de Câncer Research and Ethics Committee. Written informed consent from the parents or legal guardians were obtained from children and adolescent. All young subjects gave written informed consent in accordance with the Declaration of Helsinki. The protocol was approved by the Instituto Nacional de Câncer Research and Ethics Committee under the registry number CEP/INCA#117/12; CONEP: PB #888.2772.3.

## Author Contributions

EN conducted, supervised, and analyzed all the experiments in this study, and wrote the manuscript. LM performed mutation tests and MLPA analyses and wrote the manuscript. FA performed gene mutation tests and collected follow-up information ET-G conducted flow cytometry diagnosis. LT performed OS analyses. MP-d-O designed and supervised the study, and wrote the final version of the manuscript. All authors critically reviewed and approved the final draft of the manuscript.

### Conflict of Interest Statement

The authors declare that the research was conducted in the absence of any commercial or financial relationships that could be construed as a potential conflict of interest.
