# Corrigendum: The Profile of Immunophenotype and Genotype Aberrations in Subsets of Pediatric T-Cell Acute Lymphoblastic Leukemia

**DOI:** 10.3389/fonc.2020.640629

**Published:** 2021-02-05

**Authors:** Elda Pereira Noronha, Luísa Vieira Codeço Marques, Francianne Gomes Andrade, Luiz Claudio Santos Thuler, Eugênia Terra-Granado, Maria S. Pombo-de-Oliveira, the Brazilian Collaborative Study Group of Acute Leukemia, Carolina da Paz Zampier

**Affiliations:** ^1^ Pediatric Hematology-Oncology Program, Research Center, Instituto Nacional de Câncer, Rio de Janeiro, Brazil; ^2^ Clinical Research Division, Research Center, Instituto Nacional de Câncer, Rio de Janeiro, Brazil

**Keywords:** T-cell acute lymphoblastic leukemia, childhood, immunophenotypic subtypes, molecular alterations, early T-cell precursor acute lymphoblastic leukemia, overall survival

Dr. Marcela Mansur was erroneously included as consortium author and should be removed from the section below:

## Brazilian Collaborative Study Group of Acute Leukemia

Carolina da Paz Zampier, Thayana da Conceição Barbosa, Paulo Chagas Neto, Gisele Dallapicola Brisson, Filipe Vicente dos Santos Bueno, Ingrid Cezar Sardou, Bruno Gonçalves Aguiar, Anna Carolina Silva Dias, Patrícia Carneiro de Brito, Geraldo Pedral Sampaio, Raimundo Antônio Gomes Oliveira, Claudia Teresa de Oliveira, César Casagranda, Geni Ramos Vera, Gustavo Ribeiro Neves, Isis Maria Quezado Magalhães, José Carlos Córdoba, Juliana Teixeira Costa, Rebeca Ferreira Marques, Renata Pereira de Souza Barros, Renata Sarkis Alves, Renato Guedes, Sidnei Epelman.

In the original article, previously published data from this author was not correctly acknowledged. A correction has been made to **MATERIAL AND METHODS, T-ALL Molecular Characterization, paragraph no. 1:**

“Partial clinical, molecular and prognostic data on Brazilian samples diagnosed from 2005-2012 have been previously published (19, 20).”

There is also an error in the **Acknowledgements**. The correct names of the funders partially supporting this work are 1) TUCCA-Associação para Crianças e Adolescentes com Câncer, São Paulo, Brazil; 2) SwissBridge Fundation- Fundação do Câncer, Rio de Janeiro, Brazil (# L0J SWB 2014 SUB-PROJECT 1.A).

The authors apologize for these errors and state that they do not change the scientific conclusions of the article in any way. The original article has been updated.
