# Supplementary material for: The Profile of Immunophenotype and Genotype Aberrations in Subsets of Pediatric T-Cell Acute Lymphoblastic Leukemia
Source: Front Oncol. 2019 Apr 30;9:316. doi: 10.3389/fonc.2019.00316 (PMC6503680; doi:10.3389/fonc.2019.00316)
Supplement: Supplementary file 1 [file Table_1.DOC]

**Supplementary Table 1: Panel of monoclonal antibodies and fluorochromes used and revisited in the diagnostic panel.**

|  | FITC | PE | PerCP-Cy5.5 | PE-Cy7 | APC | APC-H7 |
| --- | --- | --- | --- | --- | --- | --- |
| First round MoAb | aMPO | cyCD22/CD79a | CD45 | CD19 | cyCD3 |  |
|  | CD7 | mCD3 |  |  | CD34 |  |
| Complementary  MoAb | CD2 | CD1a | CD45 | CD8 | CD7 | mCD3 |
|  | CD4 | CD7 |  | CD117 | CD11b |  |
|  | CD5 | CD10 |  | CD56 | CD34 |  |
|  | CD14 | CD11b |  | CD10 |  |  |
|  | CD15 | CD13 |  | HLA-DR |  |  |
|  | CD16 | CD19 |  |  |  |  |
|  | CD42 | CD33 |  |  |  |  |
|  | CD65 | CD34 |  |  |  |  |
|  | CD61 | CD41 |  |  |  |  |
|  | TCRγδ | CD64 |  |  |  |  |
|  | HLA-DR | TCRαβ |  |  |  |  |
|  | TdT |  |  |  |  |  |

Abbreviations: MoAb – monoclonal antibodies; cy – cytoplasmatic; m – membrane
