# Supplementary material for: The Profile of Immunophenotype and Genotype Aberrations in Subsets of Pediatric T-Cell Acute Lymphoblastic Leukemia
Source: Front Oncol. 2019 Apr 30;9:316. doi: 10.3389/fonc.2019.00316 (PMC6503680; doi:10.3389/fonc.2019.00316)
Supplement: Supplementary file 2 [file Table_2.DOC]

**Supplementary Table 2:** **The demographic and clinical features of pediatric T-ALL according to maturational subtypes, Brazil 2015-2017.**

|  |  |  |  | **Early Late** | | | **Mature^&^** | | |
| --- | --- | --- | --- | --- | --- | --- | --- | --- | --- |
|  | **Total** | **ETP** | **imature** | **cortical** | **cortical** | **cortical** | **DP** | **SP** | **DN** |
|  | n (%) | n (%) | n (%) | n (%) | n (%) | n (%) | n (%) | n (%) | n (%) |
| **Age (years)** |  |  |  |  |  |  |  |  |  |
| <1 | 7 (2.5) | 2 (7.4) | 1(2.6) | 0 (0) | 0 (0) | 0 (0) | 2 (5.7) | 2 (6.5) | 0 (0) |
| 1-10 | 153 (54.3) | 9 (33.3) | 19 (50.0) | 7 (46.7) | 28 (56) | 35 (66) | 18 (51.4) | 17 (54.8) | 18 (58.1) |
| ≥10 | 122 (43.3) | 16 (59.3)* | 18 (47.4) | 8 (53.3) | 22 (44) | 18 (34) | 15 (42.9) | 12 (38.7) | 13 (41.9) |
| **Sex** |  |  |  |  |  |  |  |  |  |
| Female | 72 (25.5) | 10 (37) | 12 (31.6) | 4 (26.7) | 10 (20) | 10 (18.9) | 7 (20) | 7 (22.6) | 12 (38.7) |
| Male | 210 (74.5) | 17 (63) | 26 (68.4) | 11 (73.3) | 40 (80) | 43 (81.1) | 28 (80) | 24 (77.4) | 19 (61.3) |
| **WBC x 10^9^/L** |  |  |  |  |  |  |  |  |  |
| <50 | 81 (28.7) | 13 (48.1)* | 10 (27) | 2 (13.3) | 16 (32) | 13 (24.5) | 5 (14.3) | 9 (29) | 11 (35.5) |
| ≥50<100 | 53 (18.8) | 2 (7.4) | 11 (28.9) | 3 (20) | 8 (16) | 13 (24.5) | 9 (25.7) | 5 (16.1) | 2 (6.5) |
| ≥100 | 148 (52.5) | 12 (44.4) | 17 (44.7) | 10 (66.7) | 26 (52) | 27 (50.9) | 21 (60) | 17 (54.8) | 18 (58.1) |
| **Mediastinal Mass** |  |  |  |  |  |  |  |  |  |
| Yes | 114 (40.6) | 7 (25.9) | 17 (44.7) | 8 (53.3) | 22 (44) | 25 (47.2) | 12 (34.3) | 12 (38.7) | 10 (32.3) |
| No | 165 (59.3) | 20 (74.1) | 21 (55.3) | 7 (46.7) | 28 (56) | 27 (50.9) | 22 (62.9) | 19 (61.3) | 21 (67.7) |
| Missing | 2(0.7) | - | - | - | - | 1 (1.9) | 1(2.9) | - | - |
| **CNS infiltration** |  |  |  |  |  |  |  |  |  |
| Yes | 19 (6.8) | 2 (7.4) | 1 (2.6) | 1 (6.7) | 2 (4.1) | 6 (11.3) | 2 (5.7) | 3 (9.7) | 2 (6.5) |
| No | 261 (93.2) | 25 (92.6) | 37 (97.4) | 14 (93.3) | 47 (95.9) | 46 (86.8) | 32 (91.4) | 28 (90.3) | 29 (93.5) |
| Missing | 2(0.7) | - | - | - | - | 1(1.9) | 1(2.9) | - | - |
| **Lymph node infiltration** |  |  |  |  |  |  |  |  |  |
| Yes | 195 (69.6) | 20 (74.1) | 29 (76.3) | 10 (66.7) | 34 (68) | 36 (67.9) | 24 (68.6) | 23 (74.2) | 18 (58.1) |
| No | 85 (30.4) | 7 (25.9) | 9 (23.7) | 5(33.3) | 16 (32) | 16 (30.2) | 10 (28.6) | 8 (25.8) | 13 (41.9) |
| Missing | 2(0.7) | - | - | - | - | 1(1.9) | 1(2.9) | - | - |

Abbreviations: *p<0.05; n – number of cases; WBC – white blood cell count; CNS – central nervous system; DP – CD4/CD8 double positive; SP – single positive to CD4 or CD8; DN – CD4/CD8 double negative. ^&^Two T-IV cases were not tested to CD4 and/or CD8.
