# Supplementary material for: The Profile of Immunophenotype and Genotype Aberrations in Subsets of Pediatric T-Cell Acute Lymphoblastic Leukemia
Source: Front Oncol. 2019 Apr 30;9:316. doi: 10.3389/fonc.2019.00316 (PMC6503680; doi:10.3389/fonc.2019.00316)
Supplement: Supplementary file 3 [file Table_3.DOC]

**Supplementary Table 3:** **Demographic, clinical-laboratorial and molecular features of the pediatric T-cell acute lymphoblastic leukemia cases selected to identify gene copy number aberrations.**

| **Variables** | **Total** | **MLPA performed** | **MLPA not performed** |  |
| --- | --- | --- | --- | --- |
|  | **n (%)** | **n (%)** | **n (%)** | ***p*-value** |
| **Age (years)** |  |  |  |  |
| <1 | 7 (2.5) | 1 (0.6) | 6 (5.3) | 0.050 |
| 1-9 | 153 (54.3) | 93 (55.4) | 60 (52.6) |  |
| ≥10 | 122 (43.3) | 74 (44) | 48 (42.1) |  |
| **Sex** |  |  |  |  |
| Female | 72 (25.5) | 48 (28.6) | 24 (21.1) | 0.167 |
| Male | 210 (74.5) | 120 (71.4) | 90 (78.9) |  |
| **WBC (X10^9^/L)** |  |  |  |  |
| <50 | 81 (28.7) | 43 (25.6) | 38 (33.3) | 0.013 |
| 50-100 | 53 (18.8) | 25 (14.9) | 28 (24.6) |  |
| ≥100 | 148 (52.5) | 100 (59.5) | 48 (42.1) |  |
| **Mediastinal Mass** |  |  |  |  |
| Yes | 114 (40.4) | 67 (39.9) | 47 (41.2) | 0.500 |
| No | 166 (58.9) | 99 (58.9) | 67 (58.8) |  |
| Missing | 2(0.7) | 2(1.2) | 0 |  |
| **CNS infiltration** |  |  |  |  |
| Yes | 19 (6.7) | 8 (4.8) | 11 (9.6) | 0.145 |
| No | 261 (92.6) | 158 (94) | 103 (90.4) |  |
| Missing | 2(0.7) | 2(1.2) |  |  |
| **Lymph node infiltration** |  |  |  |  |
| Yes | 195 (69.1) | 116 (69.0) | 79 (69.3) | 0.502 |
| No | 85 (30.1) | 50 (29.8) | 35 (30.7) |  |
| Missing | 2(0.7) | 2(1.2) | 0 |  |
| **T-ALL subtypes (EGIL)** |  |  |  |  |
| T-I | 7 (2.5) | 6 (3.6) | 1 (0.9) | 0.072 |
| T-II | 73 (25.9) | 51 (30.4) | 22 (19.3) |  |
| T-III | 103 (36.5) | 57 (33.9) | 46 (40.4) |  |
| T-IV | 99 (35.1) | 54 (32.1) | 45 (39.5) |  |
| **Molecular alterations** |  |  |  |  |
| *NOTCH1* mut/nt | 110/231 (47.6) | 82/168 (48.8) | 28/63 (44.4) | 0.657 |
| *FBXW7* mut/nt | 38/224 (17) | 30/168 (17.9) | 8/56 (14.3) | 0.682 |
| *IL7R* mut/nt | 16/225 (7.1) | 11/164 (6.7) | 5/61 (8.2) | 0.777 |
| *RAS* mut/nt | 19/231 (8.2) | 13/163 (8.0) | 6/68 (8.8) | 0.798 |
| *FLT3* mut/nt | 9/222 (4.1) | 6/167 (3.6) | 3/55 (5.5) | 0.693 |
| *STIL-TAL1* pos/nt | 49/231 (21.2) | 33/141 (23.4) | 16/90 (17.8) | 0.327 |
| *TLX3* pos/nt | 25/245 (10.2) | 11/152 (7.2) | 14/93 (15.1) | 0.080 |
| **Total** | **282 (100)** | **168 (59.6)** | **114 (40.4)** |  |

Abbreviations: MLPA – multiplex ligation-dependent probe amplification; WBC – White blood cell count; CNS – central nervous system; mut – mutated; nt – number tested; pos – positive.
