# Supplementary material for: The Profile of Immunophenotype and Genotype Aberrations in Subsets of Pediatric T-Cell Acute Lymphoblastic Leukemia
Source: Front Oncol. 2019 Apr 30;9:316. doi: 10.3389/fonc.2019.00316 (PMC6503680; doi:10.3389/fonc.2019.00316)
Supplement: Supplementary file 4 [file Table_4.DOC]

| Variable | pOS % (SE) | Mean* months (SE) | 95%CI | p-value |
| --- | --- | --- | --- | --- |
| Sex |  |  |  | 0.389 |
| Female *vs* Male | 68.8 (0.68) *vs* 58.9 (0.45) | 44.6 (3.40) *vs* 41.9 (1.98) | (37.9-51.3) *vs* (38.1-45.8) |
| Age |  |  |  |  |
| <1 year | 22.2 (0.19) | 22.0(9.58) | (3.2-40.8) | ***0.036*** |
| 1-10 years | 66.7 (0.52) | 45.3(2.23) | (40.9-49.6) |
| ≥ 10 years | 58.3(0.57) | 40.9(2.62) | (35.9-46.1) |
| WBC x10^9^/L |  |  |  |  |
| < 50 | 65 (0.72) | 45.4(2.99) | (39.5-51.2) | 0.335 |
| ≥50<100 | 52 (0.93) | 37.1(4.42) | (28.4-45.8) |
| ≥100 | 63.1 (0.50) | 43.1(2.29) | (38.6-47.6) |
| CNS infiltration |  |  |  |  |
| Yes *vs* No | 57.9(1.47) *vs* 62.8(0.39) | 40.5(6.87) *vs* 43.3(1.75) | (27.0-54.0) *vs* (39.8-46.7) | 0.695 |
| Lymphnodes |  |  |  |  |
| Yes *vs* No | 68.8(0.44) *vs* 49.2 (0.69) | 45.9(1.95) *vs* 36.8(3.20) | (42.1- 49.8) *vs* (30.6-43.1) | ***0.008*** |
| Mediastinal Mass |  |  |  |  |
| Yes *vs* No | 70.8(0.56) *vs* 56.7 (0.50) | 47.5(2.37) *vs* 40.1 (2.33) | (42.8-52.1) *vs* (35.5- 44.7) | ***0.044*** |
| Subtypes |  |  |  |  |
| ETP-ALL *vs* Others | 55.2 (0.12) *vs* 62.7 (0.40) | 39.9 (5.22) *vs* 43.2 (1.79) | (29.7-50.2) *vs* (39.7-46.7) | 0.588 |
| Imature *vs* Others | 78.5 (0.09) *vs* 59.5 (0.04) | 49.8 (4.10) *vs* 41.9 (1.84) | (41.8-57.8) *vs* (38.3-45.5) | ***0.109*** |
| Early cortical *vs* Others | 48.6(0.15) *vs* 63.0(0.39) | 35.4(7.19) *vs* 43.4(1.74) | (21.3- 49.5) *vs* (40.1-46.9) | 0.231 |
| Cortical *vs* Others | 68.1(0.09) *vs* 60.1(0.04) | 46.7(3.64) *vs* 41.9(1.91) | (39.5-53.8) *vs* (38.0-45.5) | 0.258 |
| Late cortical *vs* Others | 64.1(0.09) *vs* 61.0(0.04) | 44.1(3.85) *vs 4*2.4(1.89) | (36.5-51.7) *vs* (38.7-46.1) | 0.759 |
| Mature DP *vs* others | 63.0(0.10) *vs* 62.0(0.04) | 42.2(4.87) *vs* 43.1(1.81) | (32.6-51.7) *vs* (39.5-46.6) | 0.878 |
| Mature SP *vs* others | 49.4(0.13) *vs* 63.5(0.04) | 36.8(5.73) *vs* 43.6(1.76) | (25.6-48.1) *vs* (40.2-47.1) | ***0.194*** |
| Mature DN *vs* others | 38.6(0.18) *vs* 64.8(0.39) | 38.0(5.85) *vs* 43.8(1.80) | (26.6-49.5) *vs* (40.4-47.4) | 0.592 |
| Molecular alterations |  |  |  |  |
| *NOTCH1*^mut^ *vs NOTCH1*^wt^ | 67.6(0.06) *vs* 57.3(0.54) | 46.3(2.60) *vs* 39.2(2.63) | (41.2-51.4) *vs* (34.0-44.3) | ***0.069*** |
| *FBXW7*^mut^ *vs*  *FBXW7*^wt^ | 69.7(0.92) *vs* 61.6 (0.45) | 47.4(3.97) *vs* 42.0(2.14) | (39.6-55.2) *vs* (37.7-46.1) | 0.330 |
| *NOTCH1/FBXW7*^mut^ *vs NOTCH1/FBXW7*^WT^ | 67.6 (0.55) *vs* 55.3(0.62) | 46.3(2.33) *vs* 37.8(3.08) | (41.8-50.9) *vs* (31.8- 43.8) | ***0.027*** |
| *IL7R*^mut^ *vs IL7R*^WT^ | 67.1(0.13) *vs* 63.0(0.43) | 45.7(6.07) *vs* 43.4(1.95) | (33.8-57.6) *vs* (39.5-47.1) | 0.873 |
| *RAS*^mut^ *vs* *RAS*^WT^ | 81.7 (0.12) *vs* 63.7(0.04) | 52.1(5.11) *vs* 43.3(1.99) | (42.1-62.1) *vs* (39.4-47.2) | 0.243 |
| *FLT3*^mut^*vs FLT3*^wt^ | 59.3 (0.19) *vs* 62.9(0.43) | 44.7(7.25) *vs* 43.1(1.96) | (30.5-58.9) *vs* (39.2-46.9) | 0.939 |
| *STIL/TAL1*^pos^*vs STIL/TAL* ^neg^ | 47.6(0.90) *vs* 66.0(0.47) | 33.6(4.51) *vs* 45.4(2.00) | (24.8-42.5) *vs* (41.5-49.3) | ***0.006*** |
| *TLX3*^pos^ vs  *TLX3*^neg^ | 48.5 (0.15) *vs* 64.4 (0.04) | 41.8(5.24) *vs* 43.5(1.94) | (31.6-52.2) *vs* (39.6-47.2) | 0.702 |
| *CDKN2A/B^del^ vs CDKN2A/B^wt^* | 62.6(0.59) *vs* 62.6(0.85) | 43.3(2.66) vs 41.3(4.20) | (38.1- 48.5) *vs* (33.1-49.5) | 0.729 |
| *MYB* ^amp^ *vs MYB*^WT^ | 46.7(0.16) *vs* 64.0(0.51) | 31.5(8.84) *vs* 43.7(2.30) | (14.2-48.8) *vs* (39.2-48.2) | *0.113* |
| Grouping |  |  |  |  |
| Epigenetic** |  |  |  |  |
| Altered *vs* WT | 65.9(0.09) *vs* 61.2(0.57) | 43.1(4.68) *vs* 42.4 (2.59) | (33.9-52.3) *vs* (37.4-47.5) | 0.880 |
| Trancription Factors*** |  |  |  |  |
| Altered *vs* WT | 55.6(0.62) *vs* 65.9(0.67) | 39.4(2.86) *vs* 45.6(2.87) | (33.8-45.0) *vs* (40.0 - 51.3) | ***0.126*** |
| Signalling § |  |  |  |  |
| Altered *vs* WT | 67.1(0.06) *vs* 62.3(0.68) | 45.7(2.79) *vs* 41.6(3.29) | (40.2-51.1) *vs* (35.2-48.1) | 0.397 |
| Total | 62.0(0.04) | 42.7 (1.7) | (39.5-46.1) |  |

**Supplementary Table 4: T-cell acute lymphoblastic leukemia and variables in univariate analysis for overall survival, Brazil, 2005-2017.**

Abbreviation: pOS – probability of overall survival; SE – standard error, CI – confidence interval, SP – CD4 and/or CD8 simple positive; mut – mutated; WT – wild type; pos – positive; neg – negative; amp – amplified. *mean survival in months, **Epigenetic regulators: *EZH2*^del^, *SUZ12*^del^, *PHF6*^del^*,* ***Transcription Factors Grouped: *STIL-TAL1*^pos^, *TLX3*^pos^, *LEF1*^del^, *MYB*^amp^, §Signaling genes grouped: *N/KRAS*^mut^, *IL7R*^mut^, *NUP214-ABL1*^amp^, *PTEN*^del^, *NF1^del^*, *PTPN2*^del^; vs – versus.
