# Supplementary figures and images for: The Profile of Immunophenotype and Genotype Aberrations in Subsets of Pediatric T-Cell Acute Lymphoblastic Leukemia
Source: Front Oncol. 2019 Apr 30;9:316. doi: 10.3389/fonc.2019.00316 (PMC6503680; doi:10.3389/fonc.2019.00316)

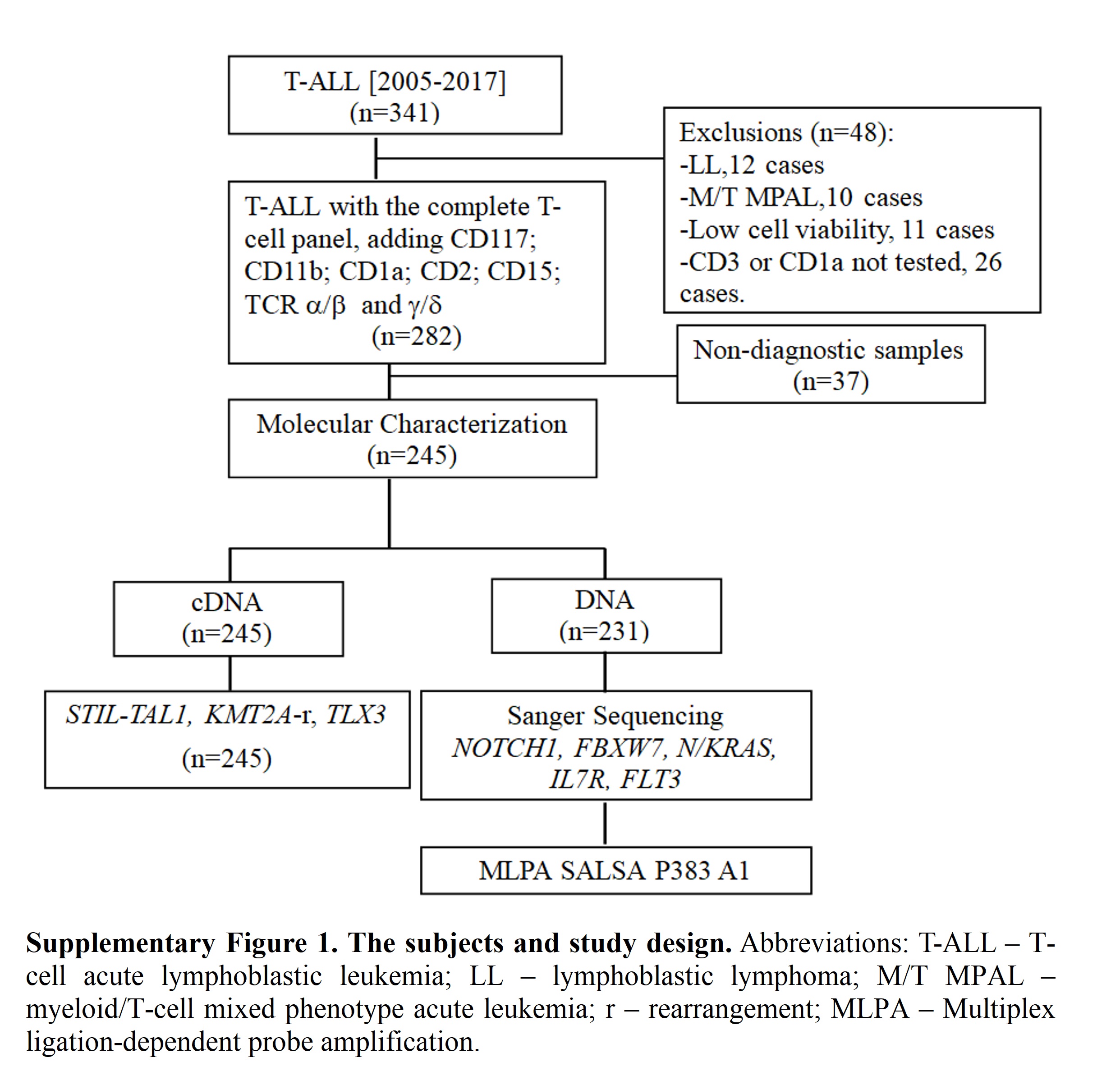

Supplement: Supplementary file 5 [file Image_1.JPEG]
