# Supplementary material for: The Profile of Immunophenotype and Genotype Aberrations in Subsets of Pediatric T-Cell Acute Lymphoblastic Leukemia
Source: Front Oncol. 2019 Apr 30;9:316. doi: 10.3389/fonc.2019.00316 (PMC6503680; doi:10.3389/fonc.2019.00316)
Supplement: Supplementary file 6 [file Image_2.pdf]

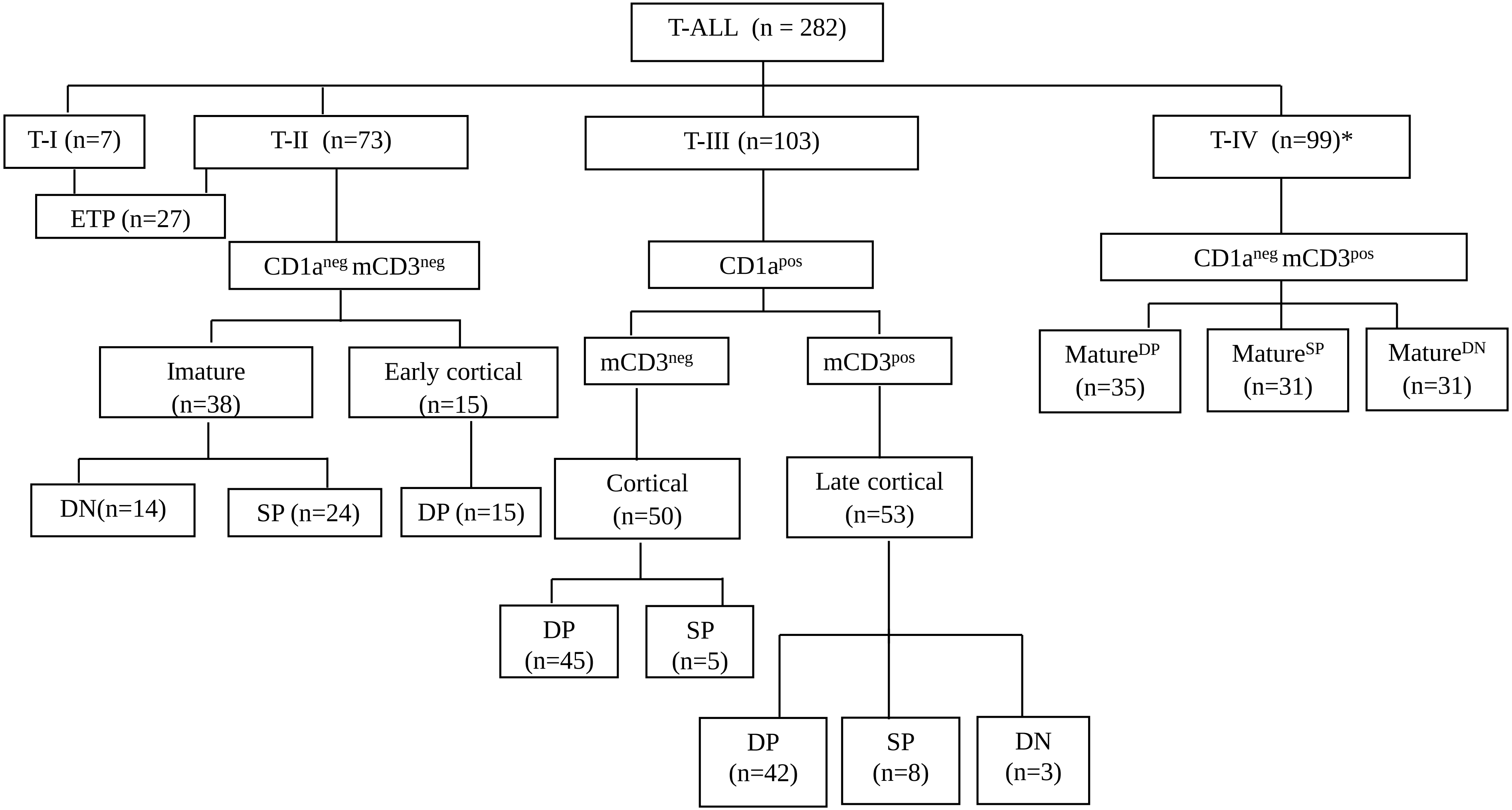

Supplementary Figure 2: Revisiting the immunophenotypic subtypes of our cohort of pediatric T-cell Acute lymphoblastic Leukemia.

Abbreviations: T-ALL – T-cell Acute lymphoblastic Leukemia; ETP – early T cell precursor; Neg – negative; Pos – positive; n – number of cases, DN – CD4/CD8 double negative; DP – CD4/CD8 double positive; SP – Single positive for CD4 or CD8; *Two T-IV cases were not tested to CD4 and/or CD8.
